# A Comprehensive Review on Non‐Thermal Technologies in Food Processing & Implementation in Different Food Industries: Limitations and Challenges

**DOI:** 10.1002/fsn3.71712

**Published:** 2026-04-13

**Authors:** Ali Raza, Hafiz Muhammad Abdullah, Muhammad Wasiq, Muhammad Usman Butt, Muhammad Afzaal, Abhayveer Singh, Rajashree Panigrahi, Mohd Asif Shah

**Affiliations:** ^1^ Department of Food Science Government College University Faisalabad Pakistan; ^2^ Centre for Research Impact & Outcome Chitkara University Institute of Engineering and Technology, Chitkara University Rajpura Punjab India; ^3^ Department of Microbiology IMS and SUM Hospital, Siksha ‘O’ Anusandhan (Deemed To Be University) Bhubaneswar Odisha India; ^4^ Department of Agriculture Economics Kardan University Kabul Afghanistan; ^5^ Division of Research and Development Lovely Professional University Phagwara Punjab India; ^6^ University Centre for Research & Development Chandigarh University Mohali Punjab India

**Keywords:** cold plasma technology, food industries, food quality, innovative technologies, non‐thermal food processing, nutritional preservation

## Abstract

Emerging technologies have gained traction in recent years, offering more benefits and potential to transform industries. As alternatives to conventional thermal treatments, non‐thermal processing technologies offer improved food safety and extended shelf life while preserving the functional and sensory properties of food products. Six important non‐thermal technologies were analyzed: cold plasma technology, high‐pressure processing (HPP), pulsed electric fields (PEF), ozonation, ionizing radiation (food irradiation), and ultraviolet light (UV‐C). Each technique has its own mechanism of action, applications in the food industry, and advantages over traditional approaches that drive industrial progress and build consumer confidence. These techniques offer environmental stability, superior product quality, and enhanced energy efficiency. However, challenges and limitations to the implications and operation remain, including scalability, regulatory obstacles, and consumer perceptions. In some cases, their effects on the nutritional value and quality of food are negligible because they are non‐thermal. Researchers should focus on the use of emerging technologies to enhance proficiency, like AI (Artificial Intelligence) and nanotechnology.

## Graphical abstract

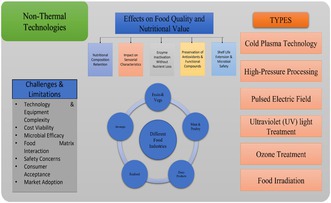

## 1 Introduction

Non‐thermal food processing technologies are increasingly replacing traditional thermal processing methods due to their ability to deliver safer, higher‐quality products with minimal nutrient loss. In response to consumer demand, these techniques enhance consumer awareness of minimally processed food products. Many thermal processing methods, such as sterilization and pasteurization, effectively inactivate microorganisms during food processing, thereby extending shelf life. However, the output effect on the food products is a loss of nutritional content and quality. For instance, thermal pasteurization can reduce total phenols by up to 76%, vitamin C by 100%, and carotenoids by 70.18% (Chiozzi et al. 2022; Mariod et al. 2024). As a result, several non‐thermal techniques have a significant impact in overcoming food safety limitations while preserving desirable product characteristics (Allai et al. 2023).

Non‐thermal technologies do not require high temperatures to operate, which prevents heat‐induced losses in food. Instead, their processes depend on physicochemical or electromagnetic principles to inactivate spoilage‐causing microorganisms (Hassoun et al. 2020). These technologies retain key nutrients more effectively than heat‐based processes, making them valuable tools for longer‐term preservation of nutritional properties (Cano‐Lamadrid and Artés‐Hernández 2022). Studies have shown that HPP can preserve more than 90% of ascorbic acid as compared to thermal processing (Abera 2019).

Common non‐thermal technologies include high‐pressure processing (HPP) (Bolumar et al. 2021), pulsed electric fields (Raso et al. 2022), cold plasma technology (Varilla et al. 2020), ionizing radiation technology (Malik et al. 2022), ozone treatment (Raghunathan et al. 2021), and ultraviolet light treatment (Chacha et al. 2021). Each technology operates via a different mechanism of action but shares the same objective of microbial inactivation while maintaining food quality (Aaliya et al. 2021). For instance, cold plasma technology generates reactive species at room temperature; pulsed electric fields enhance cell membrane permeability via electroporation; and high‐pressure processing (HPP) applies hydrostatic pressure to inactivate microorganisms (Rathod et al. 2022).

The versatility of these non‐thermal technologies allows their use across many food categories, including fruit juice, milk, vegetables, soup, eggs, fruits, and grains (Siddiqui and Chand 2022). For instance, HPP‐treated strawberry juice can have a shelf life of up to 42 days under refrigeration, compared with less than 14 days for untreated products, making HPP the optimal option for shelf‐life extension (Yildiz et al. 2021). Compared with thermal processing, non‐thermal techniques are often more sustainable and energy‐efficient, making them technologically and economically attractive (Bains et al. 2024). They help preserve bioactive compounds, including carotenoids, polyphenols, and anthocyanins (Barbosa‐Cánovas et al. 2022). Technologies such as ionizing radiation and cold plasma enhance the extraction of bioactive compounds, thereby contributing to the development of foods with added health benefits (Sruthi et al. 2022). These advantages have increased adoption among manufacturers seeking to improve yields and produce nutrient‐enriched products (Prestes et al. 2023).

Despite these benefits, non‐thermal processing still faces challenges, including scale‐up limitations, operational complexity, and the need for further optimization to ensure consistent outcomes (Lisboa et al. 2024). Current research aims to enhance efficiency, affordability, and safety to support broader global adoption (Sawale et al. 2024).

This review discusses the applications of key non‐thermal technologies, including high‐pressure processing (HPP), cold plasma technology, pulsed electric fields, ultraviolet light treatment, ozone treatment, and ionizing radiation. The mechanisms of action, applications across different food categories, and effects on safety, quality, and nutritional composition are examined. Additionally, this review outlines the challenges, limitations, and possible prospects of these technologies, emphasizing their growth in modern food processing.

## 2 Advantages of Non‐Thermal Technologies Compared to Traditional Methods

For handling all types of edibles such as meat, vegetables, fish, pulses, fruits, and spices, these non‐thermal treatments can be applied to meet the growing consumer demand for minimally processed, “clean‐label” foods. Non‐thermal methods support this demand by avoiding heat exposure, thereby helping to maintain fresh‐like sensory and nutritional qualities that consumers prefer. Over the past several decades, the food industry has largely relied on non‐thermal processing (Jadhav et al. 2021). Most notably, HPP can suppress cells of foodborne pathogens and parasites responsible for food spoilage, regardless of temperature, without disrupting flavor, texture, or color. This is because microbial inactivation occurs via pressure‐induced cellular damage rather than via heat, thereby preventing nutrient and pigment degradation (Wiśniewski et al. 2023). Cold plasma technology is an innovative process that uses high‐intensity, short‐duration gases to suppress the activity of parasites in food products. This process generates reactive species that inactivate microbes while maintaining a low product temperature, thereby preserving food quality (Feroz et al. 2019).

Traditional pasteurization extends the shelf life of food products for only a short period. It provides sustained thermal conduction to inactivate microbes in fluid foods such as juices and milk (Azizi‐Lalabadi et al. 2023). In comparison, non‐thermal methods achieve similar microbial reductions while retaining more heat‐sensitive nutrients, which are often lost during thermal pasteurization (Chiozzi et al. 2022).

**FIGURE 1:**
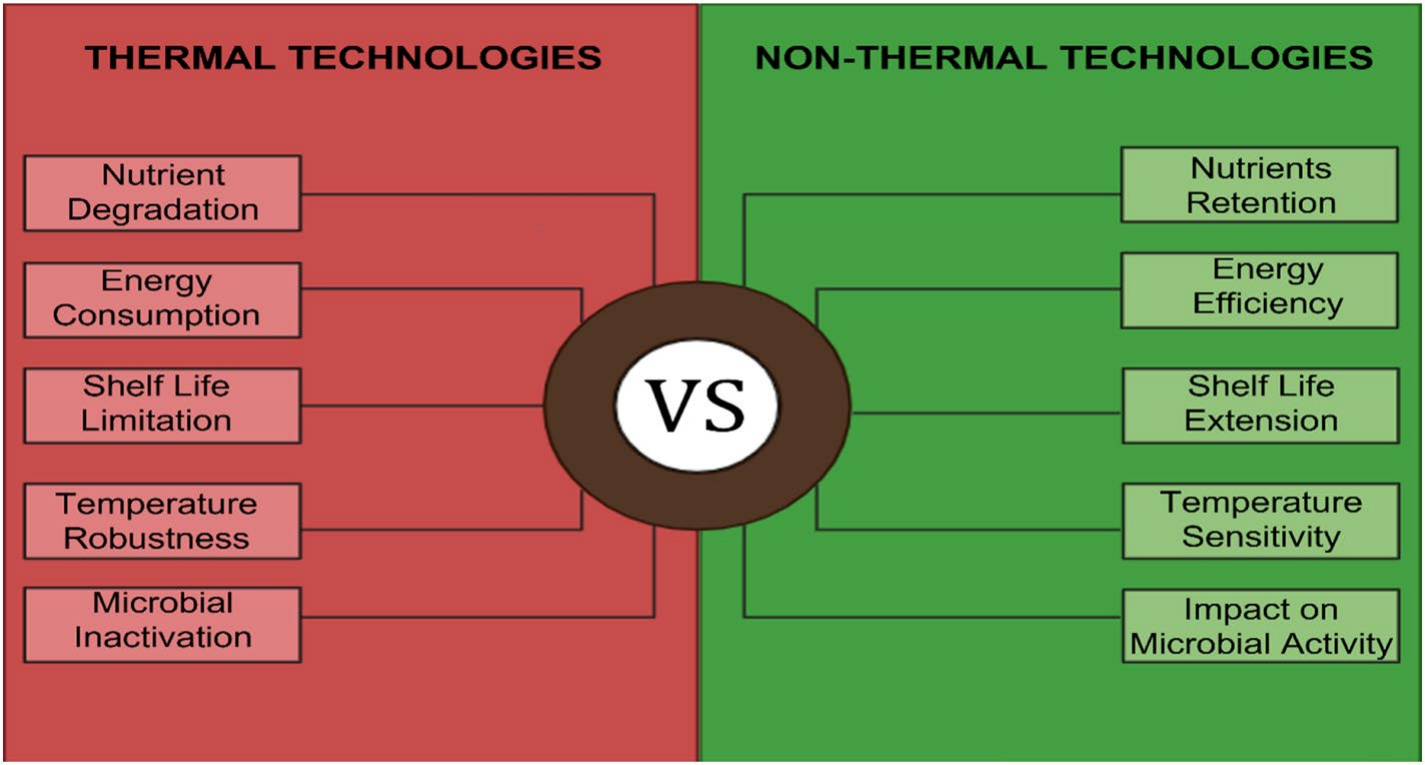
Comparison between thermal and non‐thermal technologies.

The development of microbes in foods is largely avoided because drying reduces relative humidity and water activity. The most frequently used drying techniques in the food sector are microwave‐assisted drying, atomization dryers, mechanical dryers, suspension dryers, solidification, and sun drying. These are primarily thermal drying methods that rely on the application of heat (directly or indirectly) to evaporate water. In contrast, non‐thermal drying methods such as freeze‐drying remove water via sublimation or other mechanisms without significant heat input (Figure 1). Drying techniques prevent microbial growth, as adulteration or natural decay in vegetables or fruits can lead to food spoilage and disrupt caramelization. Thermal processes are effective but often result in losses of volatile compounds and heat‐sensitive nutrients due to prolonged exposure to high temperatures (Alp and Bulantekin 2021).

Non‐thermal processing offers numerous advantages over conventional methods. It provides an emerging generation of fortified foods with preserved bioactive compounds (Ramakrishnan et al. 2023). Supercritical carbon dioxide technology has appeared as an innovative non‐thermal extraction technique with numerous benefits, such as extending shelf life, decreasing microbial density, removing the enzymes that cause the generation of non‐essential fatty acids that are responsible for the deterioration and spiced fragrance in milk, and inhibiting the internal enzymes that cause juice quality loss. In supercritical carbon dioxide technology, carbon dioxide is carried beyond its critical threshold at 7380 kPa (kilopascals) and approximately 31°C to kill the toxic enzymes. This advantage occurs because processing temperatures remain close to ambient levels, which prevents nutrient breakdown and aroma loss commonly associated with thermal treatments (Allai et al. 2023).

The traditional drying processes primarily used in the food sector are hot‐air treatment systems, which are generally regarded as high‐energy processes that increase heat‐trapping gases and account for approximately 15% of total production costs. This high energy demand is linked to continuous heating requirements, which significantly increase operational costs compared with non‐thermal processes (Menon et al. 2020). Thermal techniques have been considered energy‐intensive because they rely on heat generated by burning fossil fuels. Moreover, greater heat intensity during handling leads to depletion of volatile compounds, protein denaturation, and lower food quality standards. Meanwhile, non‐thermal techniques are effective due to their energy efficiency and ecological balance (Chakka et al. 2021).

These comparisons highlight the magnitude of differences between thermal and non‐thermal methods in terms of quality retention and energy use.

Many non‐thermal and thermal technologies are used to reduce harmful microbes that degrade fresh produce during the pre‐harvest and post‐harvest phases. Although thermal technologies are effective in achieving sufficient microbial safety, they primarily degrade the freshness and nutrient profile of the fresh food products. As a result, consumers increasingly prefer foods that offer extended shelf life, minimal nutrient loss, and better preservation of freshness. To meet these preferences, experts have evaluated various non‐thermal technologies, most of which are effective (Varalakshmi 2021).

Consequently, non‐thermal techniques are a viable alternative to thermal methods, as they help ensure stability and extended durability, thereby preventing unwanted alterations that affect the sensory attributes of nuts (Ogundipe et al. 2024). Non‐thermal techniques can achieve outcomes comparable to traditional techniques in terms of food security while preserving sensory attributes and bioactive compounds, yielding a product as close as possible to the original (Sánchez‐Bravo et al. 2022).

Nonthermal processing techniques, including pulsed electric fields, pressure‐based processing, cold plasma, and ultrasonic processing, have demonstrated potential to reduce salt content while maintaining sensory quality. This occurs because non‐thermal methods can alter microstructure and enhance salt diffusion, allowing reduced salt levels without compromising taste. However, the effective implementation of these approaches requires careful assessment of product development and consumer acceptance (Khan et al. 2024).

The meat sector faces a crucial challenge in the growth of clean‐label processed meat, as it requires sufficient active ingredients. In various meat products, nitrites are necessary ingredients that serve as microbicidal and oxidative stabilizers, making it difficult to identify a clean‐label substitute for all functional roles; however, their use raises health concerns due to the potential formation of carcinogenic nitrosamines, which IARC has linked to increased colorectal cancer risk from processed meat consumption. Another significant component that does not adhere to the pure label standards is phosphates. Phosphates are important for consistent texture and sensory attributes in various meat products, but excessive intake from additives has been associated with risks such as chronic kidney disease, cardiovascular disease, and vascular calcification (Calvo et al. 2023; Crowe et al. 2019; Delgado‐Pando et al. 2021).

With the growing popularity of pure‐label products that encourage health‐conscious consumer behavior, the food manufacturing sector is undergoing rapid change, leading to the processing, production, and distribution of products with minimal ingredients. The vision of food production with a pure label may seem simple. Extending the shelf life and meeting food product standards are challenging for processors without the use of additives, flavorings, and colorants (Singh et al. 2021).

However, the adoption of non‐thermal technologies still faces challenges, including equipment costs, scalability, and regulatory approval, which must be addressed for broader industry implementation.

## 3 Types of Non‐Thermal Technologies in Food Processing

Non‐thermal technologies are emerging as innovative solutions that play a crucial role in food processing. These technologies offer alternatives to conventional thermal treatments, which can degrade food quality, nutritional value, and taste (dos Santos Rocha et al. 2022). To enhance the shelf life of food products, non‐thermal technologies are used to inactivate microorganisms (Pravallika and Chakraborty 2022) (Table 1). The nutritional and sensory attributes of products are preserved longer (Martínez and Carballo 2021). Several non‐thermal technologies in food processing are employed; here, only a few are discussed, including cold plasma technology, high‐pressure processing (HPP), pulsed electric field (PEF), ozone treatment, ultraviolet light technology, and food irradiation (ionizing radiation) (Birania et al. 2022). To provide a clearer comparison across technologies, Table 2 summarizes their core mechanisms, applications, and primary benefits. This table illustrates which technologies are better suited for liquid‐food surface decontamination, shelf‐life extension, or high‐load microbial reduction.

**TABLE 1:** Different aspects related to non‐thermal and thermal techniques.

Aspect	Non‐thermal technologies	Thermal techniques	References
Consumption of energy	Non‐thermal processing techniques utilize less energy and processing costs	Thermal processes require greater energy for heating inputs and greater energy consumption	Vignali et al. (2022)
Retention of nutrients	Minimally processed foods preserve heat‐sensitive nutrients (bioactive compounds, vitamins)	Loss of heat‐sensitive nutrients (e.g., vitamins, bioactive compounds) is significantly increased by high‐temperature processing	Jafari and Capanoglu (2022)
Inactivation of microorganisms	Efficient inactivation of pathogens and removal of spoilage organisms without heat	Inactivates microorganisms at high temperatures	Bigi et al. (2023)
Impact on ecosystem	Less energy consumption & carbon dioxide emissions	The use of high temperatures requires high energy consumption and increases carbon dioxide emissions	Liu et al. (2020)
Safety & shelf life	Enhances safety by reducing microbial load and extending shelf life	Affects food quality for extending shelf life	Martín‐Belloso et al. (2023)
Sensory attributes	Texture, color, and flavor are maintained with high consumer acceptance	Change the food's sensory attributes due to heat	dos Santos Rocha et al. (2022)
Processing time	Short Processing Time for food preservation and microorganism inactivation	A much longer processing time is required for effective results	Kurian and Raghavan (2020)

**TABLE 2:** Mechanisms and applications of non‐thermal technologies.

Non‐thermal technology	Mechanism of action	Application in food processing	Benefits	References
Cold plasma technology	By generating reactive species (plasma)	Surface decontamination of produce	No heat damage, eco‐friendly, microbial inactivation	Cherif et al. (2023)
High‐pressure processing	Apply a pressure range of 0.1–1 kPa	Extend the shelf life of seafood, dairy, juices, and beverages	Enhance safety, improve food quality, and retain nutrients	Awasti et al. (2024)
Pulsed electric fields	Treat food between electrodes that generate high‐voltage pulses as 20–80 kV μs^−1^	Pasteurization of liquid eggs, yogurt, soups, juices, and milk	Minimal or without heat generation	Jin and Zhang (2020)
Ultraviolet light technology	UV light with wavelengths between 200 and 280 nm disrupts the genetic makeup (DNA)	Food packaging material decontamination, surface sterilization, and liquid food treatments	Eco‐friendly technique, fast treatment time, and no chemicals required	Sunita et al. (2022)
Ozonation	Use high dosages of ozone gas to oxidize cellular membranes	Sanitation and disinfection purposes, storage atmosphere, and cold storage	Effective against microorganisms, strong oxidizing agent, extended shelf life, green technology	Roobab et al. (2023); Yüceer (2023)
Ionizing radiations	Use of gamma rays or X‐rays to break bonds in DNA and inactivate microbes	Extend the shelf life of food products and reduce pathogenic microorganisms	Effectively removes pathogens, extends shelf life, and minimally affects nutrient value	Danyo et al. (2024)

### 3.1 Cold Plasma Technology

Cold plasma is a non‐thermal technology that generates an ionized gas containing reactive species that inactivate microorganisms at low temperatures (Ucar et al. 2021). In this technique, plasma is generated. It contains highly reactive species, including electrons, UV photons, and radicals. These reactive species interact with food surfaces and remove contaminants without altering the food's temperature (Mehta and Yadav 2022).

**FIGURE 2:**
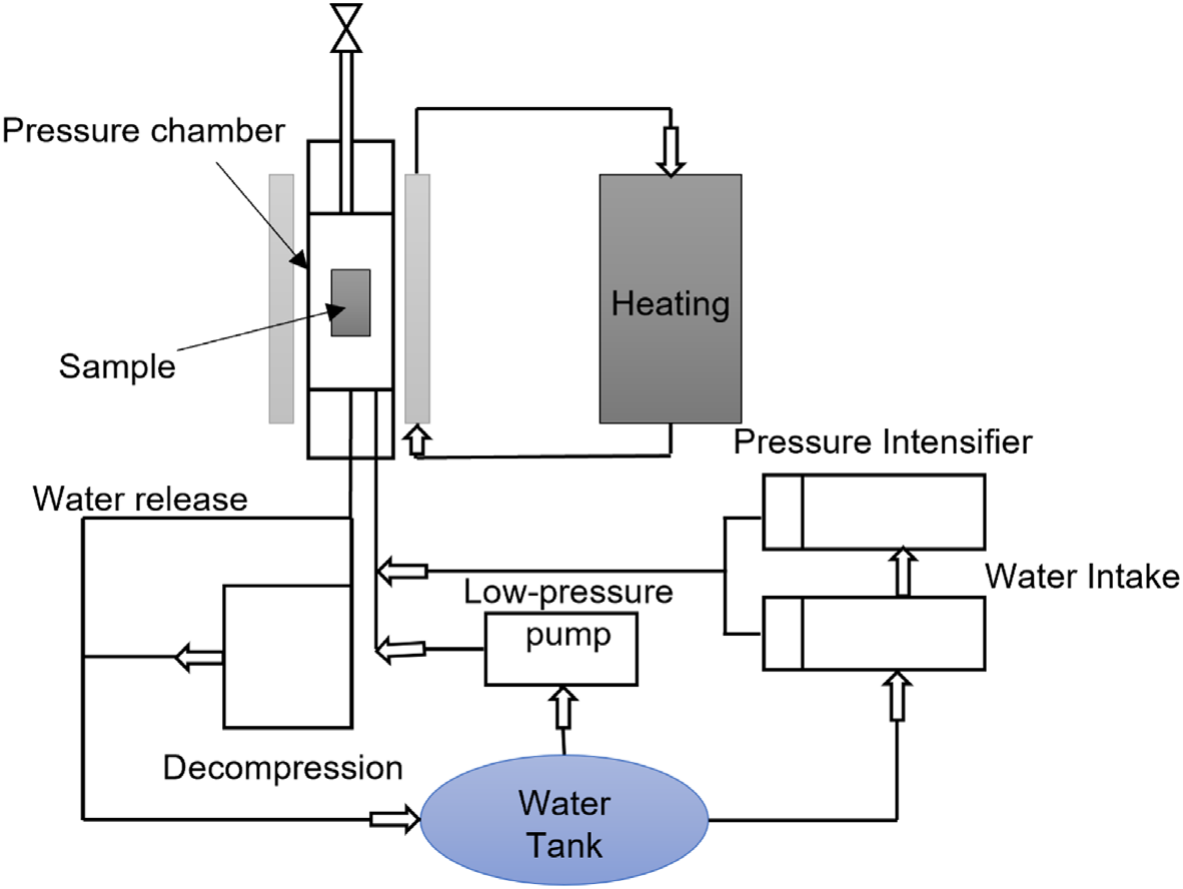
Schematic diagram of the cold plasma technique (Akhtar et al. 2022).

This technology is used to sterilize meat, food packaging materials, fruits, and vegetables with minimal impact on their structure and composition (Varilla et al. 2020). On the other hand, conventional methods rely on high temperatures and cause adverse effects (Gómez et al. 2020). Studies on APPJ (atmospheric pressure plasma jet) and DBD (dielectric barrier discharge) technologies have demonstrated significant efficacy in reducing pathogenic microorganisms, including *E. coli* and *Salmonella* (Domonkos et al. 2021). This technology can sterilize food without producing any chemical residues. It is highly beneficial for eco‐friendly, minimally processed foods (Birania et al. 2022). However, cold plasma may have limited penetration depth, making it more suitable for surface treatments (Figure 2).

### 3.2 High‐Pressure Processing

High‐pressure processing is a non‐thermal method that uses intense hydrostatic pressure, such as 0.1–0.6 k MPa, to inactivate microorganisms without applying heat (Sehrawat et al. 2021). This method does not use heat, which is why it is more suitable for sterilizing or pasteurizing food. It preserves the fresh produce, including sensory properties (texture and flavor) and nutritional composition (Amsasekar et al. 2022).

**FIGURE 3:**
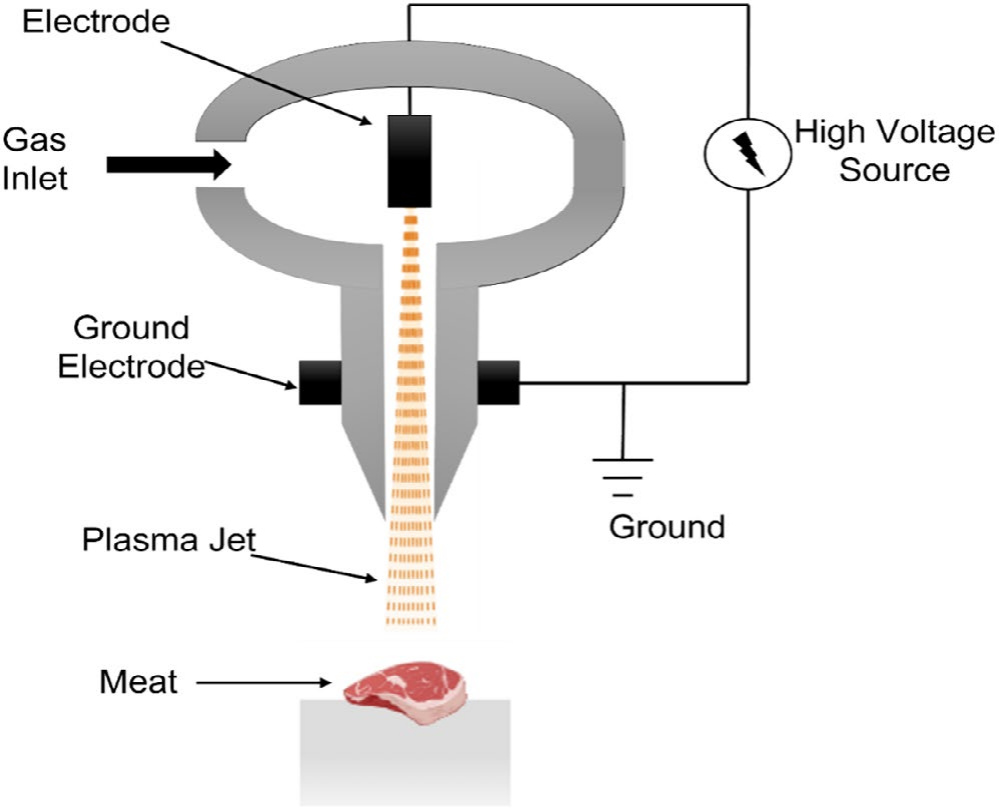
Schematic diagram of high‐pressure processing (Feroz et al. 2019).

HPP is used in the processing of many food products, such as juices, ready‐to‐eat meals, seafood products, and sauces. It is effective against pathogenic and spoilage‐causing microorganisms (Inanoglu et al. 2022). This high‐pressure, versatile method uniformly penetrates the product. The packed products are also treated after packaging to reduce the risks of contamination. However, equipment may be costly, and space for equipment placement may be limited (Silva 2023). Despite these advantages, HPP may not be suitable for low‐moisture foods and can alter the texture in some products (Figure 3).

### 3.3 Pulsed Electric Field

Pulsed electric field technology inactivates microorganisms by applying short bursts of high‐voltage electricity that disrupt cell membranes (Arshad et al. 2020). The cellular membranes of microorganisms (MOs) are disrupted by electric fields, a process known as electroporation. According to this process, cells die, and microbial inactivation is achieved (Cavalcanti et al. 2023).

It is highly efficient for many food products, such as milk, fruit juices, and smoothies, in which the distribution of a uniform electric field is ensured (Ramos‐de‐la‐Peña et al. 2020). In conventional techniques such as pasteurization, heat is used for the same purpose, whereas PEF reduces losses of food quality attributes, including nutrients, flavors, and color (Brito and Silva 2024).

**FIGURE 4:**
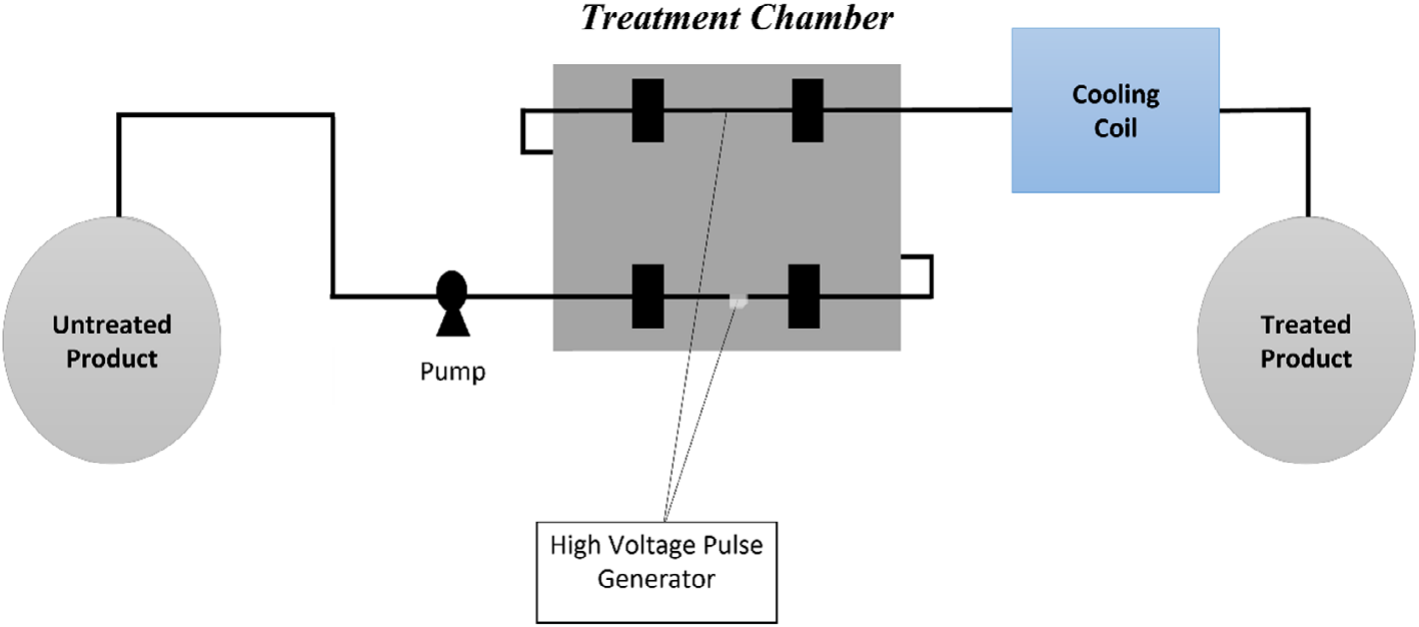
Schematic diagram of pulsed electric field (Bocker and Silva 2022).

Moreover, this technology has improved the extraction of bioactive compounds with high yield (Shiekh et al. 2021). This efficient method depends on field strength, pulse frequency, and treatment duration (Martín‐García et al. 2020). However, a challenging aspect of this field is achieving a uniform electric field distribution over a wide range (Araujo et al. 2021; Bocker and Silva 2022). PEF is less effective for solid foods because electric fields do not penetrate dense or irregular matrices effectively (Figure 4).

### 3.4 Ultraviolet Light Treatment

Ultraviolet‐C (UV‐C) radiation damages microbial DNA and prevents its replication (Dhobi 2021). This technology helps prevent the replication and growth of microorganisms (Rosario et al. 2021). Disinfection technology is expected to be implemented in the food and beverage industry (Yemmireddy et al. 2022). UV light treatment is widely used in water treatment (Iervolino et al. 2020), fruit juice sterilization, and decontamination of packaging materials (Nicolau‐Lapeña et al. 2022).

**FIGURE 5:**
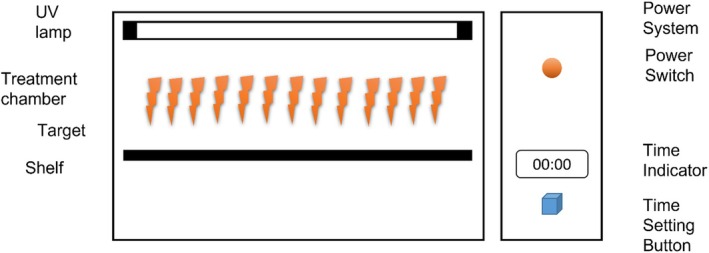
Schematic diagram of UV‐light treatment system (Bocker and Silva 2022).

Ultraviolet light technology, an energy‐efficient, chemical‐free sterilization process, inactivates pathogenic microorganisms. It is an effective method for sterilizing foods without altering sensory characteristics (Chawla et al. 2021). Its effectiveness is reduced for irregularly surfaced food products, and UV‐C can be used to process vegetables and fruits (Gómez‐López et al. 2021). Its limited penetration restricts its use for opaque or irregularly shaped foods (Figure 5).

### 3.5 Ozone Treatment

Ozone treatment relies on ozone gas, a strong oxidizing agent, to destroy microbial cell components (Dubey et al. 2022). The gaseous or dissolved form of ozone can generate reactive oxygen species (ROS). These reactive species attack microorganisms' cellular components, thereby inactivating them; however, excessive ROS production (e.g., from high ozone concentrations or prolonged exposure) may lead to undesirable oxidative damage in the food matrix, such as lipid peroxidation, protein oxidation, discoloration, off‐flavor development, or nutrient degradation (e.g., reduced ascorbic acid or lycopene content) (Sachadyn‐Król and Agriopoulou 2020; Sitoe et al. 2025).

**FIGURE 6:**
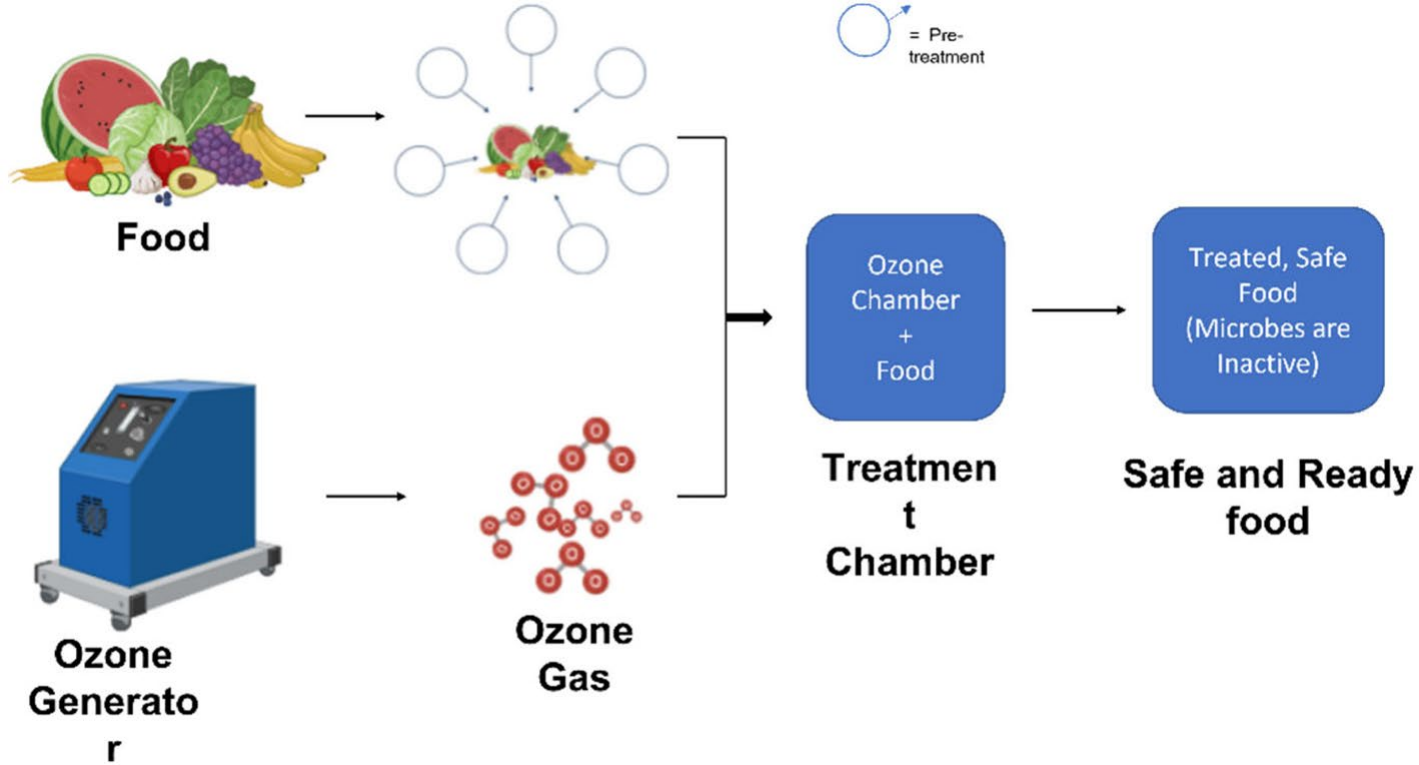
Mechanism of ozone treatment.

In the case of ozone treatment, ozone is recognized as an antimicrobial agent in food processing (Epelle et al. 2023). It is recognized as a GRAS (Generally Recognized as Safe) substance as approved by the FDS (Food & Drug Administration). Ozone has the primary advantage of being highly effective at inactivating microbes. However, this highly reactive oxygen species (O3) causes undesirable alterations, including changes in sensory properties, lipid oxidation, degradation of bioactive compounds, and loss of vitamins (Chuwa et al. 2020). Sivaranjani et al. (2021) reported that ozone reacts to form bromates as residual contaminants. Therefore, ozone must be carefully controlled to avoid quality deterioration (Figure 6).

### 3.6 Food Irradiation

Food irradiation uses ionizing radiation such as gamma rays, X‐rays, or electron beams to inactivate microorganisms in food products. These radiations are used in food sterilization to disrupt the genetic makeup of microorganisms, thereby inactivating them (Mshelia et al. 2023).

**FIGURE 7:**
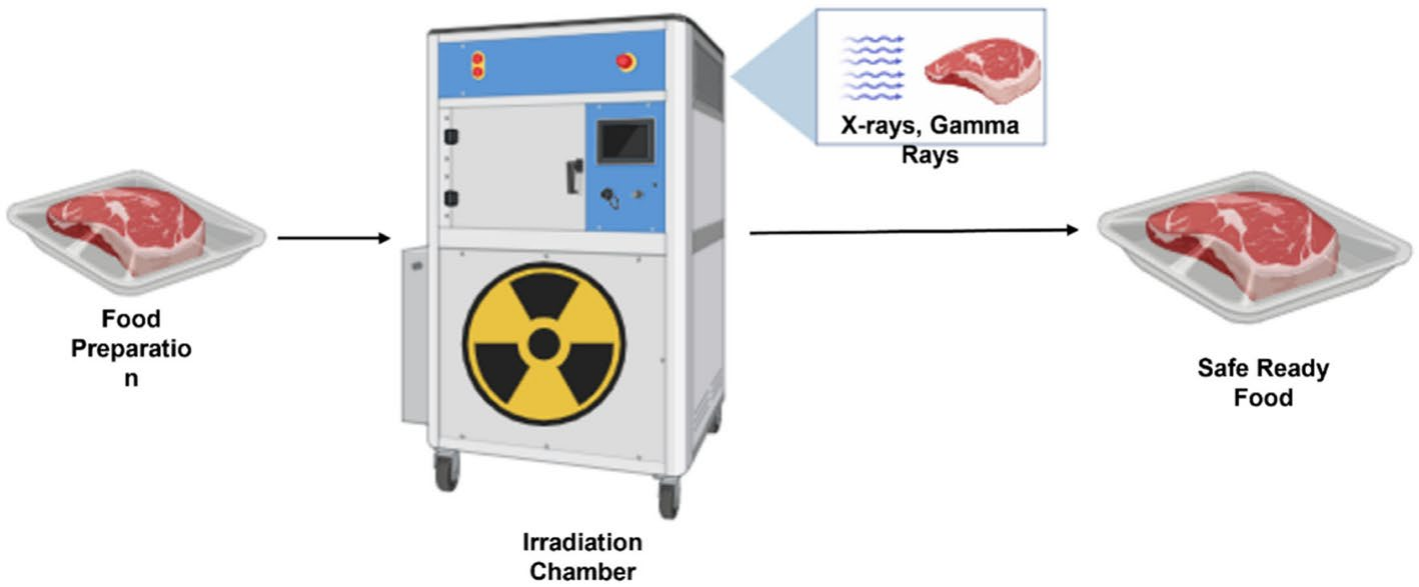
Mechanism of food irradiation.

**FIGURE 8:**
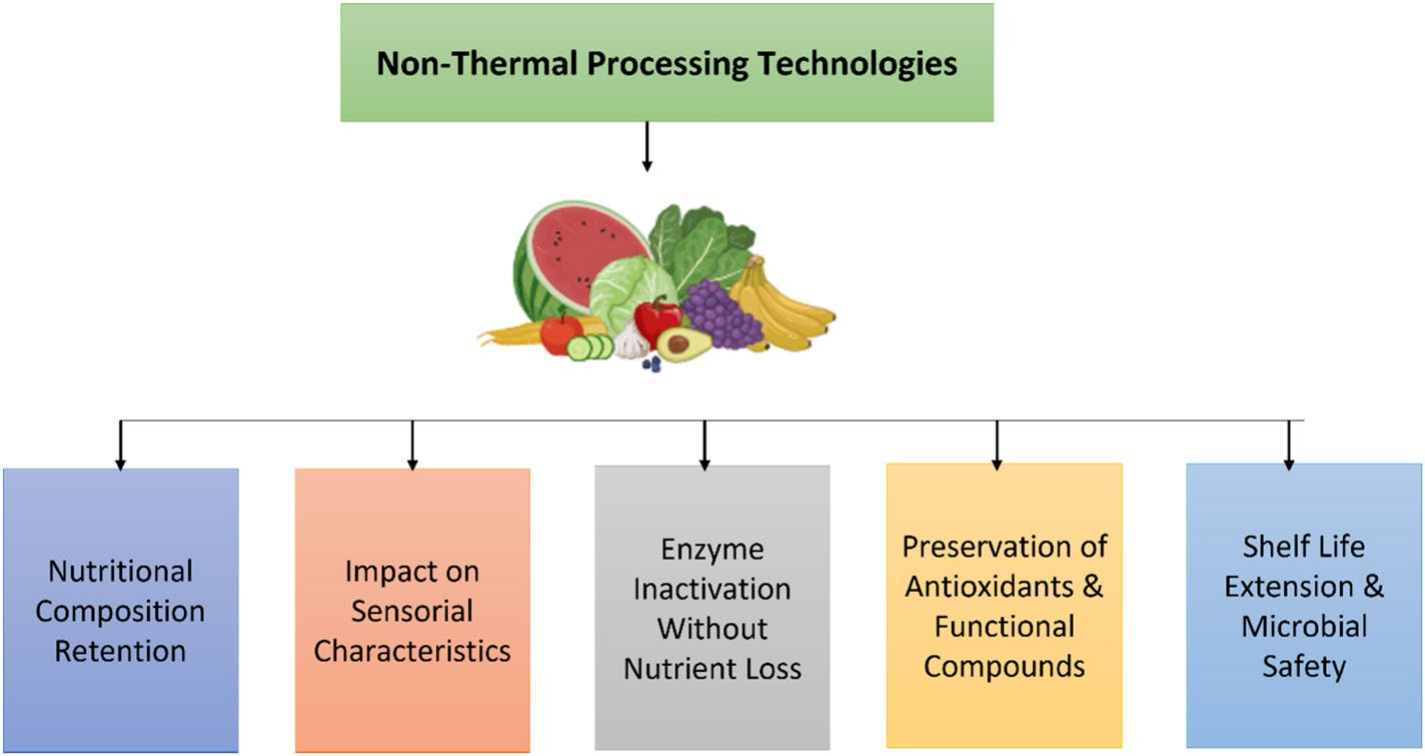
Impact of non‐thermal technologies on food.

This technique is approved by the WHO (World Health Organization), FAO, and IAEA as an effective and safe technology (Akhila et al. 2021). The use of sterilization in food packaging and medical equipment has been investigated in scientific research (Jildeh et al. 2021). Moreover, radiation does not alter the nutritional composition of food, but consumers' misunderstanding of this technique undermines its market value (Castell‐Perez and Moreira 2021). Pi et al. (2021) reported that this method of non‐thermal processing is limited by the need for multiple pieces of equipment to prevent radiation leakage, the requirement that the radiation dose exceed 10 kGy to avoid affecting the product, and the potential generation of undesirable flavor due to irradiation. Consumer acceptance remains a major limitation despite its proven safety (Figures 7 and 8).

These comparisons show that each non‐thermal technology serves specific processing goals, and selection depends on the type of food, desired quality retention, and microbial load.

## 4 Microbial Inactivation Mechanisms

Microbial inactivation without compromising product quality is achieved through non‐thermal processing techniques that disrupt microbial mechanisms, inactivate them, and ensure food safety (Lee and Yoon 2024).

### 4.1 Cell Membrane Disruption

This mechanism is among the most direct ways in which non‐thermal technologies inactivate microorganisms by targeting their structural integrity. Non‐thermal techniques like PEF and cold plasma technology are known to disrupt microbial cell membranes (Rathod et al. 2022). It involves the creation of pores in the membrane, which cause intracellular leakage of components and the cell to lose its shape. Thus, essential cellular activities are disrupted, ultimately leading to cell death (Zhang, Tan, et al. 2022). The extent of cellular membrane disruption can be changed by changing the variables such as field intensity, treatment duration, and the type of pathogen (microorganism) (Jadhav et al. 2021).

### 4.2 Mutation and DNA Damage

Another key mechanism of microbial inactivation involves direct or indirect damage to genetic material, thereby preventing replication and subsequent cellular function (Wu et al. 2020). For example, ultraviolet light induces pyrimidine dimer formation in DNA, disrupting DNA replication and transcription (Wang et al. 2023). Likewise, cold plasma generates ROS & RNS that react with cellular components, including polysaccharides, through oxidative damage. Cellular DNA is damaged, cellular functions are altered, and mutations occur due to oxidative stress produced by these species. This slows cell regeneration and proliferation. Damage to the cellular membrane causes leakage and loss of cellular function (Punia Bangar et al. 2022).

### 4.3 Denaturation of Protein

Non‐thermal technologies can inactivate microbes by disrupting protein structures, which are essential for cellular metabolism and enzyme activity. The quaternary and tertiary structures of proteins can be disrupted by using non‐thermal technologies (Rosario et al. 2021). However, the processes do not disturb functional and sensorial properties (Asaithambi et al. 2021). For example, cold plasma generates highly reactive species that oxidize amino acid side chains, thereby altering protein conformation and function (Dharini et al. 2023). This causes inactivation or damage to enzymes and other structural components, leading to metabolic dysfunction and, eventually, cell death (Wu et al. 2020).

### 4.4 Induction of Oxidative Stress and ROS Generation

Oxidative stress induced by reactive oxygen species is a central mechanism by which non‐thermal processes impair microbial function and viability. Several non‐thermal technologies create ROS, which can disrupt the components of cells like proteins, lipids, and nucleic acids. ROS are generated via several mechanisms, such as cold plasma, in which ionized gas (plasma) produces highly reactive species that interact with microbial cells (Kaushik et al. 2023). Increasing levels of reactive oxygen species overwhelm the antioxidant defense systems of microorganisms. This causes damage, and the cells lose their function, leading to cell death (Pan et al. 2020).

### 4.5 Microbial Pathway Changes & Enzymatic Activity Inhibition

Non‐thermal technologies can also target microbial metabolic pathways and enzymatic systems, disrupting essential processes required for survival and growth (Kubo et al. 2020). Technologies such as HPP and PEF have a significant detrimental effect on the enzymatic activities of cells that are important for their growth and reproduction (Yang et al. 2021). The sensory and physicochemical properties of food products can be modified by non‐thermal technologies, for instance. These biochemical changes result from cellular stress and lead to microbial death (Rosario et al. 2021).

## 5 Applications in Different Food Industries

Considerable attention is paid to non‐thermal treatments in industrial food processing. This high‐temperature‐free technology extends shelf life, ensures food safety, and maintains food quality by inactivating microbes (Adebo et al. 2021; Chiozzi et al. 2022).

### 5.1 Fruits and Vegetables

In the food and vegetable industries, non‐thermal treatments, particularly cold plasma and HPP, demonstrate exceptional efficacy in extending shelf life and enhancing food product safety (Pant et al. 2022). The HPP technique is used at high pressure to inactivate microorganisms and preserve nutrients (Amsasekar et al. 2022). On the other hand, in cold plasma technology, ionized gases are used to generate reactive species for removing surface contaminants from fresh produce. It improves safety against microbial contamination without altering fresh sensory attributes, such as texture or color (Nwabor et al. 2022).

### 5.2 Meat and Poultry

Non‐thermal techniques such as pulsed electric fields and high‐pressure processing play a crucial role in the sectors of poultry and meat industries (Ruzaina et al. 2023). In pulsed electric field (PEF) preservation, microbial cell membranes are disrupted and ultimately killed when high‐voltage pulses are applied to the target meat product (Bekhit et al. 2023). HPP effectively minimizes pathogenic microbes, prolongs shelf life, preserves nutritional composition, and retains natural attributes without requiring heat (Keyata and Bikila 2024). Non‐thermal technologies reduce and inactivate pathogenic microorganisms, thereby enhancing the shelf life of poultry products. These contribute to improving food safety while reducing the drawbacks typically associated with thermal treatments (Barroug et al. 2021).

### 5.3 Dairy Products

Non‐thermal processing technologies are significantly advantageous for dairy industries, where techniques such as high‐pressure processing (HPP) and UV‐C light technology (radiation) are applied (Neoκleous et al. 2022). HPP is used for food products such as cheese and yogurt to inactivate pathogenic microorganisms while preserving nutritional and sensory characteristics (Keyata and Bikila 2024). UV‐C light inactivates pathogens very effectively in dairy products. This technology extends shelf life and enhances safety in dairy products without the use of heat (Delorme et al. 2020). Additionally, it is used to sterilize equipment and containers (Terzioğlu et al. 2023).

### 5.4 Seafood

Non‐thermal technologies, such as cold plasma and ozone treatments, are used in seafood processing (Rathod et al. 2022; Kontominas et al. 2021). The cold plasma technique decontaminates fresh fish, enhancing food safety by targeting pathogenic microorganisms. Ozone treatment minimizes microbial load on seafood surfaces and extends the shelf life of food products (Tagrida et al. 2024). Specifically, non‐thermal technologies significantly enhance the quality and shelf life of various seafood and other products (Ekonomou and Boziaris 2021).

### 5.5 Beverages and Juices

In the juices and beverages sectors, non‐thermal technologies are gaining traction. The main non‐thermal processing methods, like high‐pressure processing (HPP) and cold plasma, are widely used (Gulzar et al. 2023). Microorganisms are inactivated by HPP technology. Depending on factors such as pressure magnitude, treatment time, temperature, sample composition (juices & beverages), microbiota, and compression and decompression rates (Podolak et al. 2020). Ready‐to‐drink food items are preferred for this technique (Huang et al. 2020). Cold plasma technology uses plasma (ionized gas) under controlled conditions to inactivate microbes, decontaminate surfaces, and extend product shelf life without altering nutritional content. Charged particles come into contact with pathogens, disrupt their cell membranes, and eliminate microorganisms. Moreover, this technology can be used in packaging to extend shelf life (Ucar et al. 2021).

## 6 Effect on Food Quality and Nutritional Value

Non‐thermal technologies in the food processing sector offer numerous benefits over conventional thermal techniques, particularly for sensory preservation, functional attributes, and nutritional value (dos Santos Rocha et al. 2022). These methods inactivate enzymes and harmful microorganisms without affecting the food through high temperatures. Non‐thermal technologies preserve the nutritional composition of fresh foods (Rosario et al. 2021).

### 6.1 Nutritional Composition Retention

Non‐thermal processing technologies can preserve essential nutrients, such as vitamins and bioactive compounds. Conventional thermal treatments, such as sterilization and pasteurization, degrade heat‐sensitive compounds, including ascorbic acid, polyphenols, and carotenoids (Barbosa‐Cánovas et al. 2022). Innovative non‐thermal advanced processing technologies, including pulsed electric fields (PEF), high‐pressure processing (HPP), and ultraviolet light treatment, sustain the strength of nutrients (Cano‐Lamadrid and Artes‐Hernandez 2021). Mechanically, HPP preserves nutrients by applying uniform pressure, which inactivates microorganisms without generating heat that degrades vitamins. Similarly, PEF creates transient pores in cell membranes that allow juice extraction and release bioactive compounds without thermal degradation (Amsasekar et al. 2022). Likewise, PEF technology can preserve anthocyanins without altering their bioavailability (Stübler 2022).

### 6.2 Impact on Sensorial Characteristics

Food quality depends on several sensory characteristics, including appearance, texture, composition, functionality, nutritional value, taste, and consumer satisfaction (Mihafu et al. 2020). Non‐thermal processing technologies are highly effective at preserving characteristics relative to conventional thermal techniques (Ucar et al. 2021). For example, cold plasma technology is known for decontaminating fresh produce without altering its flavor or texture (Zhang, Zhang, et al. 2022). PEF prevents enzymatic browning by disrupting plant cell membranes, thereby limiting contact between polyphenol oxidase and its substrates. HPP maintains texture in seafood and meat by preserving the native protein structure while inactivating spoilage organisms (Roobab, Chacha, et al. 2022; Brito and Silva 2024).

### 6.3 Enzyme Inactivation Without Nutrient Loss

Non‐thermal processing technologies are also essential for maintaining and controlling the enzymatic activities in food products without changing the nutritional composition (Jadhav et al. 2021). Enzymes such as peroxidase and polyphenol oxidase can cause undesirable alterations, such as browning, in fruits and vegetables. These enzymes are inactivated in non‐thermal conditions during processing (Basak and Chakraborty 2022). For example, cold plasma generates reactive oxygen and nitrogen species that oxidize amino acids in polyphenol oxidase, thereby inactivating the enzyme while preserving vitamins and bioactive compounds (Umair et al. 2022).

### 6.4 Preservation of Antioxidants and Functional Compounds

Potentially active compounds, such as flavonoids and polyphenols, are important for the health‐promoting qualities of foods (Di Lorenzo et al. 2021). Non‐thermal technologies in food processing preserve these bioactive compounds (Ali et al. 2021). For instance, HPP effectively increases the extraction of bioactive compounds by cellular structure disruption (Huang et al. 2020). This occurs because PEF and HPP disrupt cell walls and membranes, thereby facilitating the release of intracellular antioxidants, polyphenols, and flavonoids, thereby improving their bioavailability (Lončarić et al. 2020; Mieszczakowska‐Frąc et al. 2021).

### 6.5 Shelf‐Life Extension and Microbial Safety

Non‐thermal processing technologies are designed to inactivate microorganisms, thereby extending the shelf life of food products and improving their quality. These technologies do not affect the natural state of products (Shabbir et al. 2020). For example, cold plasma technology eliminates pathogens from the surfaces of fresh produce without compromising food quality (Ucar et al. 2021). Mechanistically, HPP uniformly inactivates microorganisms by disrupting cell membranes, whereas cold plasma generates reactive species that oxidize microbial cellular components. Both methods preserve the food matrix and sensory qualities, thereby extending shelf life without thermal damage (Khaliq et al. 2021; Khouryieh 2021).

## 7 Challenges and Limitations

Recently, non‐thermal technologies have gained significant attention due to their ability to preserve food quality, extend shelf life, and enhance microbial safety without the adverse effects of heat treatments. These technological food‐processing treatments help maintain sensory characteristics and, most crucially, nutritional value. The preservation of key attributes, including color, texture, and taste, makes these methods ideal for minimally processed or fresh foods (Barbhuiya et al. 2021).

Despite numerous advantages, non‐thermal processing technologies face numerous challenges and limitations that hinder their broad acceptance in the food industry (Kubo et al. 2023). Dangal et al. (2024) reported that non‐thermal processing may be constrained by technical limitations, financial constraints, scaling issues, and regulatory challenges. Another important limitation may be the interaction between non‐thermal treatments and food matrices, as well as consumer acceptance.

### 7.1 Technological Limitations and Equipment Complexity

Precise control over the necessary parameters is crucial at each step of the non‐thermal technique. Equipment and processing conditions are tailored to specific requirements to achieve efficient results, including superior food quality, through microbial inactivation (Kubo et al. 2020). For instance, high‐pressure processing employs specialized vessels capable of withstanding high pressures (Patel and Patel 2023). A Pulsed Electric Field (PEF) requires an advanced pulse generator, chamber, and electrodes (Naliyadhara et al. 2022). On the other hand, cold plasma technology requires carefully controlled environmental conditions and settings to generate and use plasma uniformly (Birania et al. 2022).

Moreover, food industries face technical challenges when scaling up, particularly with non‐thermal technologies, whereas small‐scale laboratories require minimal investment. Industrial‐scale production requires high costs and framework adjustments. This may impede the adoption of innovations such as non‐thermal technologies for large‐scale manufacturing, particularly in budget‐constrained sectors of the food industry (Zhang et al. 2019).

### 7.2 Cost & Economic Viability

The investment required to install non‐thermal technologies at a large scale in the food industry is a major financial challenge (Bigi et al. 2023). Equipment for implementing these novel technologies can be costly, both in capital and operating costs. The energy and maintenance requirements contribute to the overall production cost (Roobab, Fidalgo, et al. 2022).

For instance, HPP requires generating high pressure to create the necessary conditions (Huang et al. 2020). PEF requires higher costs related to the power consumption and generation of pulses (Arshad et al. 2020). Consequently, these factors result in higher production and selling prices for final products than for conventionally processed foods, potentially limiting immediate accessibility in price‐sensitive markets and not directly eliminating food security challenges through affordability alone. Moreover, ROI (return on investment) can be slow to materialize when implementing these technologies (Arya et al. 2023).

However, the technologies offer long‐term benefits for consumers and broader food systems, including superior retention of fresh‐like qualities, extended shelf life (reducing food waste and post‐harvest losses), enhanced food safety without chemical additives, and improved nutritional/sensory value, contributing indirectly to food security by increasing the availability of safe, nutritious, and minimally processed foods over time, particularly as equipment costs decline with scale and technological advancements (Bigi et al. 2023; Arshad et al. 2022).

### 7.3 Limited Microbial Efficacy & Food Matrix Interaction

Microbial inactivation is a fundamental concern of non‐thermal technologies; however, these technologies are not effective against all harmful bacteria and spoilage microbes. Some bacterial spores are not affected by non‐thermal technologies and may need more interventions for complete inactivation (Lv et al. 2021). For instance, HPP can inactivate the vegetative form of bacteria but is ineffective against spore forms. However, when combined with other useful methods, such as mild heat or the addition of preservatives, it works against spores (Aldrete‐Tapia and Torres 2021). Likewise, the cold plasma method may be less effective against certain biofilms on complex surfaces (Rao et al. 2020). Moreover, the food matrix's interaction with non‐thermal technologies can change the effectiveness against microbial inactivation (Aaliya et al. 2021). Factors affecting the efficacy of non‐thermal treatments include pH, fat content, water activity, and food composition (Barbhuiya et al. 2021). For example, the effect of PEF on microorganisms is limited by the high fat content in some products, which provides a protective barrier (Oey et al. 2022).

### 7.4 Regulatory and Safety Concerns

Regulatory aspects of non‐thermal technologies in food processing are still evolving, creating challenges for their implementation. In several regions, non‐thermal processes are subject to stringent regulatory inspections to ensure safety (Alsaleem et al. 2021). For instance, cold plasma processing technology generates reactive species to interact with food matrices and form new compounds (Cheng et al. 2020). Moreover, regulatory authorities assess the safety, quality, and effectiveness of non‐thermal technologies used in food processing. This requires time and can be costly for food manufacturers (Varalakshmi 2021) (Table 3).

**TABLE 3:** Challenges and limitations faced by non‐thermal technologies.

Non‐thermal technology	Challenges	Limitations	References
Cold plasma technology	It is difficult to treat large volumes of food, and reactive species degrade its quality	Penetration limitation in large‐volume foods may alter sensory characteristics	Sruthi et al. (2022)
High‐pressure processing	Interaction and maintenance of pressure and food composition	Its effectiveness against spores is limited	Balasubramaniam (2021)
Pulsed electric field	Non‐uniform distribution of the electric field in the food	Not very effective for inactivating bacterial spores	Bocker and Silva (2022); Timmermans et al. (2022)
Ultraviolet light treatment	Less efficient in non‐clarified juices, and surface irregularities can reduce efficacy	Limited the penetration of certain food products	Ramos et al. (2024); Delorme et al. (2023)
Ozone treatment	Ozone instability can lead to the formation of unwanted compounds in food	Limited penetration into certain foods and potential exposure to harmful (carcinogenic) chemicals, such as bromates	Aslam et al. (2021); Sivaranjani et al. (2021)
Food irradiation	Consumer misconceptions about nuclear technology use If ambient temperature is not used, it can cause heat‐sensitive nutrient loss	High equipment requirements increase technology costs and exacerbate inadequate sanitization. Limited consumer acceptance	Jadhav and Choudhary (2024); Joshua Ajibola (2020)

### 7.5 Consumer Acceptance and Market Adoption

Consumer perceptions remain a fundamental barrier to the adoption of products developed using non‐thermal technologies. Several users may be unfamiliar with non‐thermal processing procedures and may also have concerns about the efficiency and safety of these technologies. Such as misunderstandings about the use of cold‐plasma food‐processing methods and their potential association with hazardous compounds. Further studies are needed to raise consumer awareness of the benefits of non‐thermal technologies. As a result, organizations need to manage the technological benefits of non‐thermal techniques as they are required to earn consumer trust and belief (Silva et al. 2024).

## 8 Innovations and Trends in Non‐Thermal Technologies

Non‐thermal technologies in the food industry have gained significant traction due to their ability to preserve nutrients, extend shelf life, improve food quality, and ensure food safety. Healthier, minimally processed foods are increasingly popular among consumers (Nonglait et al. 2022). Advanced sectors in non‐thermal processing technologies are needed to focus on high‐impact applications, innovations, and implications for food quality and safety (Pipliya et al. 2023).

### 8.1 Combination of Multiple Non‐Thermal Technologies

Synergistic effects from integrating diverse non‐thermal technologies are a prominent trend (Boateng 2023). For example, when UV‐C (254 nm) is combined with cold plasma technology, it can enhance microbial inactivation while preserving the nutritional and sensory qualities of food products. Researchers integrate technologies to enhance the efficiency and efficacy of food preservation, thereby offering a comprehensive approach to food safety (Kulawik et al. 2023).

### 8.2 Plasma Technology Advancements

Another significant advancement in non‐thermal processing technology is cold plasma, which focuses on improving plasma generation techniques and expanding its use in food processing (Sonawane and Patil 2020). Recent developments have introduced more controllable and efficient methods for inactivating microorganisms and preserving food for longer. Studies indicate that the use of plasma‐activated water as a sanitizing agent for washing fresh fruits and vegetables (produce) has broad applications in the food sector (Sharma et al. 2021).

### 8.3 Automation & Real‐Time Monitoring

Automation and real‐time monitoring effectively characterize the future of non‐thermal technologies. The food industry is expanding, with larger sectors emerging. The use of up‐to‐date technologies is essential in non‐thermal processing systems, enabling optimal results through precise control of processing parameters and conditions (Režek Jambrak et al. 2021). Collecting real‐time data can ensure quality and improve compliance with food safety standards, thereby enhancing consumer trust and product stability (Kyaw et al. 2024).

### 8.4 Novel Packaging Solutions Development

Another area that may be important for the growth of non‐thermal technologies is innovation in packaging materials. Smart and active packaging solutions can only leverage non‐thermal processing technologies to enhance the shelf life and safety of food products (Gabrić et al. 2022). For instance, antimicrobial properties in packaging materials are introduced when the material is treated with cold plasma. This reduces the risk of contamination and increases the shelf life of food products (Perera et al. 2022). Moreover, smart packaging incorporating sensors to detect sensory changes is important for ensuring food integrity (Pou et al. 2022).

### 8.5 Focus on Energy Efficiency and Sustainability

Concern about eco‐friendly methods is increasing to reduce resource consumption and minimize pollution, while food preservation is maximized (Subaitha et al. 2024). For example, the utilization of renewable resources in plasma generation has become a key trend in food processing methods (Sonawane and Patil 2020). Future innovations and trends will focus on a more sustainable environment, as consumers have recommended. It also contributes to global efforts to reduce waste and increase environmental maintenance (Jadhav et al. 2021).

## 9 Conclusion and Future Trends

Non‐thermal technologies have seen significant development in food processing, offering effective alternatives to traditional methods. These methods do not compromise the quality, safety, or nutritional value of food products. Microbial safety and retention of sensory characteristics are additional advantages of non‐thermal processing technologies. The future of these emerging technologies in the food industry appears promising, given the increasing consumer demand for minimally processed and fresh foods. Manufacturers and researchers should focus on optimizing conditions and discovering synergistic combinations to improve their performance and applications. Additionally, advances in automation and real‐time monitoring could facilitate the integration of these technologies into commercial production lines by improving efficiency and consistency. As the food industry grows, it prioritizes sustainable, pollution‐free ecosystems and food safety. They should not compromise on the future of healthier and safer food systems. Positive collaboration among researchers, regulatory bodies, and industry stakeholders can meet global food requirements by ensuring high standards of food safety and quality.

## Author Contributions

**Ali Raza:** writing – original draft (equal). **Muhammad Afzaal:** validation (equal). **Hafiz Muhammad Abdullah:** validation (equal). **Muhammad Wasiq:** software (equal). **Muhammad Usman Butt:** formal analysis (equal). **Abhayveer Singh:** visualization (equal). **Rajashree Panigrahi:** validation (equal). **Mohd Asif Shah:** software (equal), formal analysis (equal).

## Funding

The authors have nothing to report.

## Conflicts of Interest

The authors declare no conflicts of interest.

## Data Availability

Although all the available data provided in the manuscript can be obtained from authors on request basis.
